# Modulation of Swine Gut Microbiota by Phytogenic Blends and High Concentrations of Casein in a Validated Swine Large Intestinal In Vitro Model

**DOI:** 10.3390/vetsci10120677

**Published:** 2023-11-27

**Authors:** Igor V. Popov, Naiana Einhardt Manzke, Mônica Maurer Sost, Jessica Verhoeven, Sanne Verbruggen, Iuliia P. Chebotareva, Alexey M. Ermakov, Koen Venema

**Affiliations:** 1 Centre for Healthy Eating & Food Innovation (HEFI), Maastricht University—Campus Venlo, 5928 SZ Venlo, The Netherlands; i.popov@maastrichtuniversity.nl (I.V.P.); m.maurersost@maastrichtuniversity.nl (M.M.S.); s.verbruggen@maastrichtuniversity.nl (S.V.); 2 Faculty of Bioengineering and Veterinary Medicine, Don State Technical University, 344000 Rostov-on-Don, Russiaaermakov@donstu.ru (A.M.E.); 3 Division of Immunobiology and Biomedicine, Center of Genetics and Life Sciences, Sirius University of Science and Technology, Federal Territory Sirius, 354340 Sochi, Russia; 4 Animal Health Concepts BV, 8141 GN Heino, The Netherlands; n.manzke@ahcbv.com; 5 Division of Nanobiomedicine, Center of Genetics and Life Sciences, Sirius University of Science and Technology, Federal Territory Sirius, 354340 Sochi, Russia

**Keywords:** TIM-2, in vitro colon model, 16S rRNA, metagenomics, SCFA, phytogenic blends, pig

## Abstract

**Simple Summary:**

The wide use of antibiotics as growth promoters poses a great threat to One Health, as the acquisition of resistance to antibiotics in bacteria leads to ineffective therapy of infectious diseases. That is why there is a need for alternatives for antibiotic growth promotors. Phytogenic blends are composed of various plant-derived bioactive compounds and are considered an alternative to antibiotics as feed supplements for improving production performance due to their beneficial effects on swine gut microbiota, resulting in improving the overall wellbeing of pigs. In this study, we used an artificial swine large intestine model to assess how two phytogenic blends and their combinations with casein affect swine gut microbiota. As a result, we observed that a combination of phytogenic blends and casein had the most promising effect as modulators of microbiota composition, as their supplementation caused an increase in the abundance of bacteria, which are associated with greater production performance in pigs. The results of this study showed promising feed additives for pig diet as replacement of antibiotics as growth promotors, which could have beneficial effects on growth performance, as the microbiota composition of pigs is directly correlated with it. However, the data should be proven in living pigs, as artificial GIT models do not fully reproduce the swine physiology.

**Abstract:**

Phytogenic feed additives are gaining popularity in livestock as a replacement for antibiotic growth promotors. Some phytogenic blends (PB) positively affect the production performance, inhibit pathogens within the gut microbiota, and improve the overall health of farm animals. In this study, a swine large intestine in vitro model was used to evaluate the effect of two PBs, alone or in combination with casein, on swine gut microbiota. As a result, the combination of casein with PB1 had the most beneficial effects on swine gut microbiota, as it increased the relative abundance of some commensal bacteria and two genera (*Lactobacillus* and *Oscillospiraceae* UCG-002), which are associated with greater production performance in pigs. At the same time, supplementation with PBs did not lead to an increase in opportunistic pathogens, indicating their safety for pigs. Both PBs showed fewer changes in swine gut microbiota compared to interventions with added casein. In contrast, casein supplementation significantly increased beta diversity and the relative abundance of commensal as well as potentially beneficial bacteria. In conclusion, the combination of casein with PBs, in particular PB1, had the most beneficial effects among the studied supplements in vitro, with respect to microbiota modulation and metabolite production, although this data should be proven in further in vivo studies.

## 1. Introduction

Phytogenic blends, also known as phytochemicals, phytobiotics, or simply phytogenics, are composed of various plant-derived bioactive compounds that can be used as feed additives for livestock animals. These blends are recognized for their antioxidant, anti-inflammatory, and antimicrobial properties, which contribute to enhanced growth performance and overall positive effects on the health and well-being of farm animals [1,2,3]. Given their antimicrobial effectiveness, phytogenic blends have recently gained attention as potential alternatives to antibiotic growth promoters [4].

The efficacy of phytogenics as feed additives in swine production is influenced by a range of factors. These include the specific composition and dosage of the phytogenics, the encapsulation technology employed for their protection and delivery, as well as potential synergistic interactions with other feed additives or antibiotics [5]. For example, Nowak et al. studied the combination of phytogenics with probiotics and prebiotics [6], while Duarte et al. investigated the combined use of phytogenics, composed of carvacrol and thymol, with antibiotic [7]. Both studies demonstrated positive outcomes from phytogenics alone, but not in combination with other additives in the diets when measuring performance and overall gut health in pigs. At the same time, Diao et al. showed a synergic effect of a phytogenic compound (thymol) and benzoic acid on the performance and gut microbiota composition of pigs [8].

Numerous studies have shown that the key to the beneficial effects of phytogenics on swine health and production performance lies in their ability to modulate the gut microbiota. This is crucial because the composition and activity of intestinal microbes are closely linked to intestinal morphology, the immune system, and gastrointestinal physiology [9,10,11]. One efficient and convenient method for studying the modulation of swine gut microbiota by various feed additives involves the use of artificial gastrointestinal systems. These systems are designed to replicate the conditions found in different parts of the gastrointestinal tract (GIT) [12]. In this study, we used the TIM-2-based validated Swine Large Intestinal in vitro Model (SLIM) [13] to investigate the impact of casein and two distinct phytogenic blends on pig microbiota composition, as well as SCFA and BCFA production.

## 2. Materials and Methods

### 2.1. Study Product

Phytogenic blend 1, phytogenic blend 2 (PB1 and PB2, Animal Health Concepts, Heino, The Netherlands), and casein were added to the standard ileal efflux medium (SIEM) for the SLIM experiments (Table 1). SIEM was included as a control condition and consisted of starch, pectin, xylan, arabinogalactan, amylopectin, protein, vitamins, salts, Tween 80, and ox bile, as described by Cuevas-Tena et al. [14]. Both phytogenic blends contained a mix of essential oils and herbal extracts, including components found in rosemary, turmeric (curcuma), cinnamon, red pepper, and oregano oil in different proportions.

### 2.2. Fecal Samples Collection and Preparation

Grab samples, but only fresh feces and not contaminated with urine, were collected from the floor from growing pigs (48 pens with 6 pigs/pen; Hypor Libra × Hypor Maxter, Hendrix Genetics, Boxmeer, The Netherlands) as described before [13]. The pigs received no antibiotics, and their body weight was approximately 70 kg. Pigs were fed ad libitum with a commercial diet, which was formulated to meet or exceed the nutrient requirements of growing pigs according to CVB (Dutch research institute for livestock feed and nutrition). 

To create a standardized microbiota inoculum, we homogenized fecal samples in an anaerobic cabinet (Sheldon Lab—Bactron IV, Gomelius, OR, USA) [13]. Then we snap-froze the obtained fecal slurries in 30 mL portions in liquid nitrogen and stored them at −80 °C. Before inoculation, we thawed 4 tubes of 30 mL of fecal slurry each for 1 h at 37 °C and then subsequently mixed them with pre-reduced dialysate to a total volume of 250 mL.

### 2.3. Experimental Set up and the TIM-2

We inoculated the standardized microbiota pool (Section 2.2) and 60 mL of pre-reduced dialysate to each SLIM unit. Subsequently, SIEM was administered to each unit (2.5 mL/h) for an adaptation period of 16 h. Thereafter, the units were continuously supplemented for 72 h with a constant flow of SIEM (2.5 mL/h) in conditions described in Table 1. Every 24 h, lumen samples of 25 mL were removed from the units to simulate passage from the proximal to the distal colon. Lumen and dialysate samples were obtained at 0, 24, 48, and 72 h, and analyzed for metabolite production and microbiota composition (Section 2.4 and Section 2.5).

### 2.4. Gut Microbiota Composition

Sequencing of amplicons of the V3–V4 region of the 16S rRNA gene was performed to determine microbiota compositions as described before [14]. In short, from the lumen samples, DNA was extracted, amplified with barcoding, pooled, and subsequently sequenced with the Illumina MiSeq sequencing system according to the manufacturer’s instructions (Illumina, Eindhoven, The Netherlands). The Binary Base Call text-based format for storing biological sequences and corresponding quality scores pipelines (BCL2FASTQ, v. 1.8.3, Illumina, San Diego, CA, USA) was used to convert the sequences into the FASTQ files after quality checking. Subsequently, Quantitative Insights Into Microbial Ecology 2 (QIIME-2, v. 2023.2) software was used to analyze the results [15]. For the data analysis we performed denoising with DADA2 [16], and used the Silva database (version 138 (available online: https://www.arb-silva.de/documentation/release-138, accessed on 23 January 2023)) as a reference 16S rRNA database for the classification of amplicon sequence variants [17]. Alpha diversity metrics (Shannon’s index [18], Chao1 index [19], and Pielou’s evenness [20]) and the Bray–Curtis beta diversity distance matrix [21] were also calculated.

### 2.5. SCFA and BCFA Production

Short-chain fatty acid (SCFA) and branched-chain fatty acid (BCFA) concentrations were analyzed through gas chromatography–mass spectrometry (GC–MS). Lumen samples were centrifuged at 14,000 rpm for 10 min. The clear supernatant of lumen samples (150 μL) and dialysate samples (300 μL) were mixed with internal standard solution (550 μL and 400 μL, respectively) containing methanol (Sigma, St. Louis, MO, USA), formic acid (20%; Emsure), and 2-ethyl butyric acid (Sigma). Five μL of the mixture was injected on a GC column (8890 GC Systems, Agilent, Middelburg, The Netherlands) by an automatic sampler. Concentrations of metabolites acetate, propionate, iso-butyrate, butyrate, iso-valerate, valerate, and caproate were determined by comparing obtained values from a calibration curve. Production was calculated by multiplying concentrations with the respective volumes of fractions. The number of metabolites at time point 0 h was artificially set at zero. Concentrations of the experiments with SIEM were considered as control, but were not subtracted from the results with the interventions.

### 2.6. Statistical Analysis

The programming language R (v4.2.3, R Foundation for Statistical Computing, Vienna, Austria) was used for statistical analyses. The MaAsLin2 package was used for differential abundance analysis [22]. Supplementation with SIEM only was used as a reference factor for the linear model analysis. Before differential abundance analysis, we performed filtering by applying a minimal 10% threshold for the prevalence of the taxa to get more robust results [23]. Based on acquired counts data, relative abundances of taxa were calculated. The Kruskal–Wallis test was performed to determine differences in the alpha diversity matrix. PERMANOVA with the adonis function from the “vegan” package was used to determine differences in beta diversity distances (the number of permutations was set to 1000). Non-metric multidimensional scaling (NMDS) ordination and matrix plots were used for the visualization of beta diversity distances. The correlation of taxa relative abundance and microbial metabolites was calculated with Spearman’s correlation. Multiple comparisons were adjusted with the Benjamini–Hochberg false discovery rate, and *p*-values were considered significant at *p* < 0.05 where appropriate. Adjusted *p*-values (*q*-values) were considered significant at *q* < 0.1. Visualization of acquired data and results was carried out with the “ggplot2” package.

## 3. Results

### 3.1. Changes in Microbiota Composition

**Figure 1:**
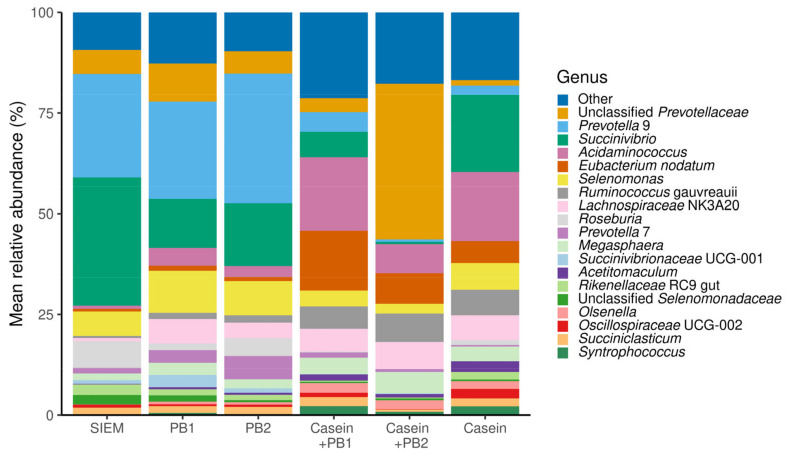
Composition in the relative abundance of identified taxa at the genus level in samples from the Swine Large Intestinal in vitro Model after interventions. SIEM = standard ileal efflux medium, phytogenic blends 1 and 2 = PB1 and PB2.

As the used swine microbiota inoculum was standardized by pooling [24], mostly the same bacterial phyla, classes, orders, families, and genera were observed among units supplemented with different feed additives throughout the experiment. Figure 1 represents the microbiota composition in the relative abundance at the genus level among compared interventions throughout the experiment. Figure S1 represents the same data for phylum, class, order and family levels. Figure S2 shows the initial microbiota composition before the supplementation of any studied blends.

**Figure 2:**
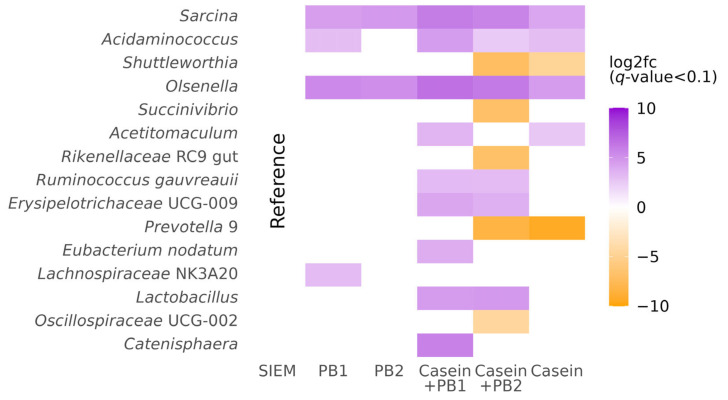
Results of differential abundance analysis at the genus level using MaAsLin2 [22]. The heatmap represents a statistically significant log2-fold change (log2fc) of taxa in SLIM units supplemented with phytogenic blends 1 and 2 (PB1 and PB2), casein, and their combinations compared to control—standard ileal efflux medium (SIEM).

According to the results of differential abundance analysis of filtered data, there were sixteen genera of which the relative abundances differed significantly between the applied interventions (Figure 2): *Sarcina*, *Shuttleworthia*, *Acidaminococcus*, *Succinivibrio*, *Catenisphaera*, *Acetitomaculum*, *Olsenella*, *Ruminococcus._gauvreauii*, *Prevotella* 9, *Rikenellaceae* RC9 gut, *Eubacterium nodatum*, *Lachnospiraceae* NK3A20, *Oribacterium*, *Oscillospiraceae* UCG.002, and *Lactobacillus*. Figure S3 contains a visualization of differential abundance analysis results for taxa at the phylum, class, order, and family levels. Figures S4–S8 represent a direct comparison of the relative abundance of these taxa.

**Figure 3:**
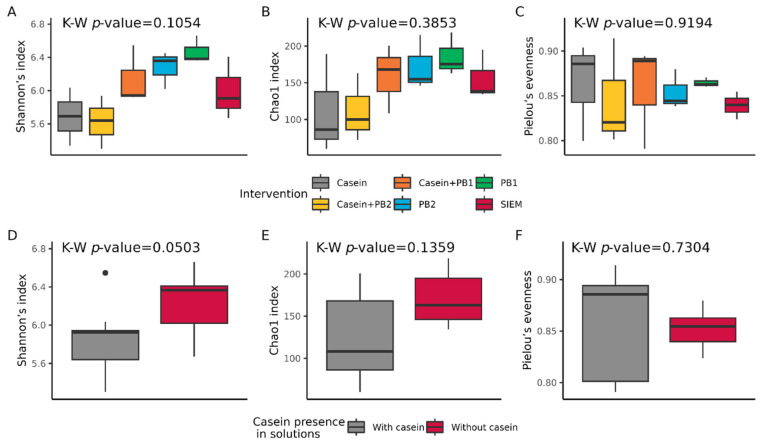
Alpha diversity metrics in samples from the in vitro model: Shannon’s index (**A**,**D**), Chao1 index (**B**,**E**), and Pielou’s evenness (**C**,**F**). Box plots A, B, and C represent alpha diversity metrics in samples from Swine Large Intestinal in vitro Model after interventions with standard ileal efflux medium (SIEM), phytogenic blends 1 and 2 (PB1 and PB2), casein + PB1, casein + PB2, or casein. Box plots D, E, and F represent alpha diversity in the same samples grouped according to the presence or absence of casein in interventions. The *p*-values were calculated with the Kruskal–Wallis (K–W) test.

There were no statistically significant differences in alpha diversity metrics between samples grouped by used feed additives and casein interventions in SLIM (Figure 3).

**Figure 4:**
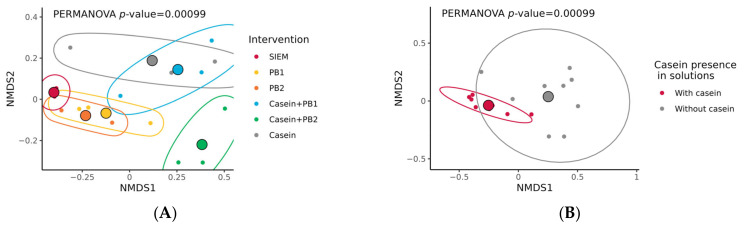
Non-metric multidimensional scaling (NMDS) ordination plot illustrating differences in beta diversity among samples from the Swine Large Intestinal in vitro Model based on Bray–Curtis distance matrix: (**A**) NMDS plot with samples grouped according to intervention: standard ileal efflux medium (SIEM), phytogenic blends 1 and 2 (PB1 and PB2), casein + PB1, casein + PB2, and casein; (**B**) NMDS plot with the same samples, but grouped according to the presence of casein in treatments. The *p*-values were calculated with the PERMANOVA test; the number of permutations was set to 1000. The large colored spheres represent centroids of the groups.

However, the beta diversity of studied microbiota based on Bray–Curtis dissimilarity was significantly different both in samples grouped by intervention (*p* < 0.001) and the presence of casein (*p* < 0.001). Figure 4 shows an NMDS ordination plot with distances between groups. The same data are visualized in the matrix plot (Figure S9), which corresponds to NMDS plots.

### 3.2. Production of SCFA and BCFA

**Figure 5:**
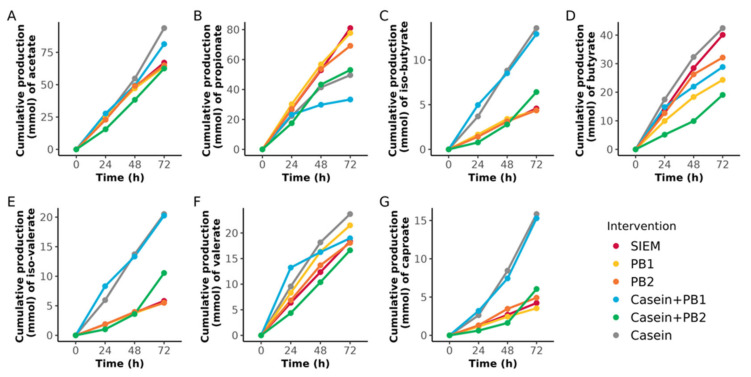
Cumulative production (mmol) of organic acids acetate (**A**), propionate (**B**), iso-butyrate (**C**), butyrate (**D**), iso-valerate (**E**), valerate (**F**), and caproate (**G**) after interventions with standard ileal efflux medium (SIEM), phytogenic blends 1 and 2 (PB1 and PB2), casein + PB1, casein + PB2, and casein.

The cumulative production of acetate, butyrate, valerate, and caproate and the BCFAs iso-butyrate and iso-valerate were the highest in SLIM units supplemented with casein after 72 h of the experiment (Figure 5), while these values were slightly lower for interventions with SIEM, PB1, and PB2. Similar values as for casein were obtained for casein + PB1 for the cumulative production of acetate, iso-butyrate, iso-valerate, and caproate. Supplementation with SIEM, PB1, and PB2 resulted in the highest values of propionate, which were above 60 mmol, whereas interventions containing casein resulted in propionate lower than 60 mmol. At the same time, the cumulative production of iso-butyrate, iso-valerate, and caproate after supplementation with SIEM, PB1, and PB2 did not get beyond 5 mmol. As expected, interventions with casein increased BCFA production. However, the addition of PB2 to casein led to a lower production of BCFAs (Figure 5C,E), which was also the case for caproate (Figure 5G).

**Figure 6:**
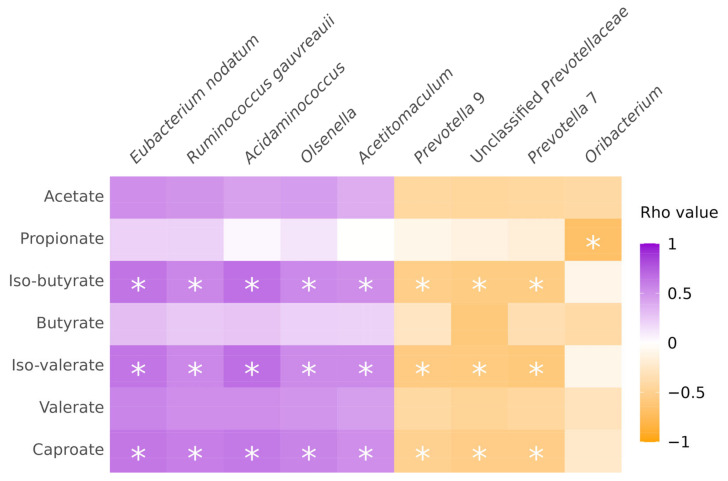
Correlation between metabolite production and specific operational taxonomic unit at the genus level for rho values ≥0.75 or ≤−0.75. White asterisks (*) *q*-value < 0.1; purple: positive correlation; orange: negative correlation.

Spearman correlations were performed between metabolite concentrations and OTUs at the genus level (Figure 6). Five taxa (*Eubacterium nodatum* and *Ruminococcus gauvreauii* groups, *Acidaminococcus*, *Olsenella*, and *Acetitomaculum*) had significant positive correlations (*q*-value < 0.1) with iso-butyrate, iso-valerate, valerate, and caproate production. Significant negative correlations (*q*-value < 0.1) were also discovered between three taxa identified as *Prevotellaceae* and iso-butyrate, iso-valerate, and caproate cumulative production. Also, the relative abundance of *Oribacterium* and propionate cumulative production had a significant negative correlation (*q*-value < 0.1).

## 4. Discussion

In this study, we assessed the effects of two phytogenic blends (PB1 and PB2), and their combination with casein on swine gut microbiota in an artificial, dynamic, computer-controlled in vitro large intestine system. Artificial GIT systems are intended to mimic the intestinal physiology of humans and animals, which makes them suitable models for pre-clinical and in vitro studies of the digestibility of foods, feed additives, and drugs and their effects on gut microbiota [25,26,27]. Validated artificial GIT models allow for conducting controlled, reproducible, and cost-effective studies, which sometimes are not achievable with in vivo models due to unpredictable factors, such as infections, uncontrolled animal welfare violations, and death of laboratory animals. Also, using artificial GIT systems complies with the principles of ethics in the use of animals in pre-clinical research, as it can reduce the number of animals used in further studies [28]. TIM-2 is a validated, dynamic computer-controlled in vitro model of the human colon [12]. For the current study, we used the TIM-2-based SLIM, which closely reproduces the large intestine physiological conditions of pigs, such as anaerobic environment, peristalsis, pH, temperature, and growth medium for gut microbes. This system was validated in a recent experiment [13].

Standardized pooled microbiota samples and SIEM were inoculated into all SLIM units before the supplementation of studied interventions. Inoculation of standardized microbiota into all units provides an opportunity to mechanistically study changes in the microbial balance as all units start with essentially the same microbiota (Figure S2) [24,29]. Figure 1 and Figure S1 show the relative abundance of taxa at different levels. All the major taxa are present in all units with different treatments, however, the relative abundance of some is significantly different among the treatments, as discussed below.

In these experiments, the microbial composition of the control SLIM unit was the most stable, and microbial diversity was less prominent in comparison to the units with applied interventions, as shown in the NMDS ordination plot (Figure 4A) and the corresponding matrix with beta diversity (Figure S9). These results additionally substantiate the effectiveness of the SLIM model in maintaining the composition of the swine gut microbiota throughout the dynamic experiment with this model [13,30,31,32].

We performed a differential abundance analysis of 16S rRNA sequencing data after filtering by removing rare taxa by applying a minimal 10% prevalence threshold. According to the results of a bioinformatical benchmark study by Nearing et al., differential abundance analysis of filtered relative abundance data provides more robust results in comparison to the analysis of the unfiltered data [23]. Also, we used MaAsLin2 for the differential abundance analysis, as it showed the most consistent results in this bioinformatical benchmarking study [23]. 

One of the greatest changes in relative abundance was observed in *Prevotella* 9, which is considered a keystone taxon in pigs. *Prevotella* has a key role in swine gut microbiota composition and functions and positively affects growth performance, the immune system, and overall health in pigs [33,34,35]. In our study, casein and a combination of PB2 and casein significantly decreased the relative abundance of *Prevotella* 9 in comparison to the control, while the combination of casein and PB1 did not have the same effect. However, it is worth mentioning that another genus identified as *Prevotella* 7 and an unclassified genus belonging to the *Prevotellaceae* family did not have significant differences. Also, a decrease in the relative abundance of the *Prevotellaceae* family was significantly associated with casein supplementation without any combinations (Figure S3D). These results generally correspond to in vivo studies, as Rist et al. showed that bacterial numbers of the Bacteroides–Prevotella–Porphyromonas group were lower in pigs fed with casein in comparison to soybean meal [36]. *Succinivibrio* is a commensal taxon in the colon and cecum in pigs and has the fiber-degrading potential [37,38,39]. The high relative abundance of this genus is also associated with better production performance parameters [40]. In a study by Shili et al., a low-protein diet increased the relative abundance of *Succinivibrio* in the gut microbiota of living pigs [41]. In our study, the inclusion of casein did not change the relative abundance of this genus; however, the addition of PB2 to casein decreased the relative abundance of *Succinivibrio*, which matches the results of the in vivo study of Shili et al. [41]. *Prevotella* and *Succinvibrio* are known for the production of propionate [42,43], and the lower cumulative production of this SCFA corresponds to the lower prevalence of *Prevotella* and *Succinvibrio* in SLIM units supplemented with casein-containing interventions (Figure 5). However, there is no significant correlation between propionate production and the relative abundance of *Prevotella* and *Succinvibrio* (Figure 6). All interventions are associated with the presence of *Sarcina*. This genus is not inherently pathogenic; however, in some studies, the presence of *Sarcina* was associated with gastric bloating-like syndrome in animals [44,45]. *Shuttleworthia* is another typical swine gut bacteria, which is mainly associated with a lowly fermentable diet [46]. In our study, as expected in view of this, the relative abundance of *Shuttleworthia* showed lower values in SLIM units supplemented with casein and casein-containing PB2 intervention. *Rikenellaceae* RC9 gut group is considered a protective gut bacteria, with high relative abundance presence in pigs corresponding to greater growth performance [47]. A combination of PB2 with casein resulted in the lowest relative abundance values of *Rikenellaceae* RC9 in the SLIM experiment, while casein alone did not affect this value compared to the control. *Acidaminococcus*, *Olsenella*, *Acetitomaculum*, *Ruminococcus gauvreauii*, *Erysipelotrichaceae* UCG-009, the *Eubacterium nodatum* group, and *Lachnospiraceae* are considered part of the normal swine gut microbiota [48,49,50,51,52,53,54,55]. In our study, all interventions except for supplementation with PB2 significantly increased the relative abundance of *Acidaminococcus*. Also, all interventions were associated with a higher relative abundance of *Olsenella* compared to SIEM. *Acetitomaculum* showed a higher relative abundance in SLIM units supplemented with casein and casein-with PB1, while the relative abundance of *Ruminococcus gauvreauii* and *Erysipelotrichaceae* UCG-009 was increased by combinations of casein and both phytogenic blends. Supplementation with PB1 in combination with casein resulted in a statistically significant increase in the relative abundance of *the Eubacterium nodatum* group, while supplementation only with PB1 increased the relative abundance of *Lachnospiraceae* NK3A20. The *Eubacterium nodatum* group is recognized for its ability to produce SCFAs and is found in higher concentrations in piglets with normal body weight compared to those with low body weight [54]. The *Lachnospiraceae* family is also well known for SCFA production. The abundance of this taxa was significantly increased in gut microbiota of piglets fed with oregano essential oil [56]. Hou et al. showed that *Catenisphaera* was responsible for the production of inflammatory cytokines in piglets [57]. In our in vitro study, a high relative abundance of *Catenisphaera* compared to control was significantly associated with supplementation with a combination of PB1 and casein. *Lactobacillus* is considered a beneficial genus for swine gut microbiota and overall health, as it is known for the promotion of growth performance and immune modulation of these farm animals [58,59,60]. Combinations of casein and both phytogenic blends significantly promoted the relative abundance of *Lactobacillus* in our study, which indicates a possible positive impact of these phytogenic additives/supplements. Moreover, several in vivo studies showed that essential oils increased the abundance of *Lactobacillus*, which corresponds to our results [61,62]. The last taxon with significant differences in the relative abundance among the studied interventions is *Oscillospiraceae* UCG.002, which had the lowest values in the microbiota from the SLIM unit supplemented with a combination of casein and PB2. The dominance of the genus UCG-002 (family *Oscillospiraceae*) in swine intestine microbiota is usually associated with higher values of growth performance in growing piglets [63,64].

In previous simple batch incubation experiments, both blends have been shown to inhibit potential pathogenic species, like *E. coli*, *Salmonella* Enteritidis, *Salmonella* Typhimurium, *Enterococcus cecorum*, *Clostridium perfringens,* and *Campylobacter jejuni* isolates from poultry and *E.coli*, *Streptococcus suis*, and *Brachyspira hyodysenteriae* isolates from pigs (data not shown). However, these simple batch incubation experiments do not necessarily translate to a complex community such as the swine gut microbiota, with all the interactions that take place in such a complex community. In this study, after differential abundance analysis, there were fifteen genera, the relative abundance of which differed significantly among studied interventions (Figure 2 and Figure S4). Although none of the genera belonging to the above-mentioned bacteria were affected by the studied phytogenic blends, there was an effect on other bacteria in the complex swine gut community, which shows their biological effect. We speculate that in order to prove the previously investigated antimicrobial effect of PB1 and PB2 against *E. coli*, *Streptococcus suis*, and *Brachyspira hyodysenteriae* within the complex swine gut microbiota community, a study should be conducted using artificial GIT units with a recreated microbiota that has known high relative abundances of these pathogens. This approach would enable us to assess the presence of these pathogens in three conditions: intact swine microbiota, infected microbiota, and microbiota treated with PB1/PB2 in an in vitro setting. In the study reported here, we found that it was not possible to assess these effects. This limitation arose because the pooled microbiota samples used for SLIM units lacked the presence of *Esherichia-Shigella*, *Streptococcus* and *Brachyspira*, Additionally, *Enterococcus*, *Campylobacter*, and *Salmonella* were either undetectable or present in only a few samples, which led to their exclusion from the analysis (Figure S2). Alternatively, examining the microbiota of animals at different life stages, such as during or around the (pre-)weaning period, could be valuable. The microbiota of animals at these stages are more likely to contain larger relative abundances of these pathogenic taxa [65]. Similarly, focusing on the microbiota of the small intestine, where these taxa are also more prevalent, could provide additional insights [65].

We also should mention that PB1 and PB2 had different effects on swine gut microbiota composition, which is probably related to different proportions of essential oils in their composition. It is known that different concentrations and even origins of phytochemicals differently affect swine health [66]. For example, in a study by Rebucci et al., treatment of weaning piglets with carvacrol, thymol, and cinnamaldehyde did not result in improvements in body weight throughout the experiment [67], while in a study by Grilli et al., a blend of thymol, vanillin, and organic acids in the diet of weaned pigs improved their growth [68]. So, considering the results of these and our studies it is clear that different compositions of phytogenic blends have different effects on swine microbiota composition, which should be taken into account in the further development of phytogenic blend-based feed additives for piglets.

There were no significant differences in the alpha diversity of swine gut microbiota supplemented with studied interventions (Figure 3). Nevertheless, there were significant differences in the beta diversity (Bray–Curtis dissimilarity), which are primarily caused by supplementing high concentrations of casein to SLIM units. The beta diversity displayed in NMDS ordination plots (Figure 4) clearly shows that casein supplementation results in larger Bray–Curtis distances between samples, while samples without casein are more ordered and stable. Dowley et al. show similar results, as they did not observe any significant effects of a casein-containing diet on alpha diversity in the gut microbiota of piglets, although beta diversity was significantly affected [69]. Gao et al. obtained the opposite results, as in their in vivo experiment a diet with a high concentration of casein resulted in significantly lower alpha diversity compared to the control, but there were no differences in beta diversity [70]. In our study, there was a tendency in decreasing the alpha diversity with casein-containing supplements (both with and without PBs), while with phytobiotics alone there is a trend for an increase, but these differences were not significant. Further in vitro or in vivo testing with a higher number of biological replicates could prove that studied phytogenic blends can promote microbial diversity, while a high concentration of casein could promote the presence of specific bacteria and decrease alpha diversity, as is shown in other in vivo studies.

Casein-containing interventions also affected cumulative SCFA and BCFA production. Supplementation with casein alone resulted in high values of almost all fatty acids except for propionate, with the highest values of iso-butyrate, iso-valerate, and caproate (Figure 5), which are considered to be fermentation products from protein fermentation. A combination of casein and phytogenic blends also resulted in high values in the cumulative production of these fatty acids, although the combination with PB2 resulted in lower iso-butyrate, iso-valerate, and caproate cumulative production. On the other hand, propionate production was the lowest in casein-containing interventions, which could be associated with decreasing propionate-producing bacteria. SCFAs and BCFAs play an important role in host–microbiota interaction, as these microbial metabolites have a lot of biological functions and effects on pigs’ health [71]. SCFAs, particularly butyrate, are energy substrates for colonocytes, promote intestinal cell proliferation, suppress pro-inflammatory cytokines, and regulate lipid metabolism, which overall results in improving the growth performance and meat quality of the pigs [9,10]. BCFAs have less prominent effects; however, they play an important role in impairing gut barrier function and regulation of pro-inflammatory cytokines [10,72]. Among SCFA and BCFA-producing bacteria in swine gut microbiota, there are the designated *Ruminococcaceae*, *Ruminococcus*, *Lachnospiraceae*, *Blautia*, *Roseburia*, *Lactobacillaceae*, *Clostridium*, *Eubacterium*, *Faecalibacterium*, *Bifidobacterium*, *Propionibacterium*, *Streptococcus*, and *Bacteroides* [67]; however, in our study not all of these taxa had a significant correlation with cumulative SCFA or BCFA-production (Figure 6). Overall, casein increased the cumulative production of acetate, iso-butyrate, iso-valerate, and caproate, which for acetate could have beneficial effects in living pigs, but for the BCFAs could result in negative effects, as these are commonly considered to be less healthy, although this should be tested in vivo to draw final conclusions.

## 5. Conclusions

In this study, we tested the in vitro effects of two phytogenic blends (PB1 and PB2), with or without a high concentration of casein in the diet, on the swine gut microbiota using a validated computer-controlled artificial swine large intestine system. Most beneficial effects on the swine gut microbiota in vitro were observed in the SLIM unit supplemented with a combination of casein and PB1. This intervention increased the relative abundance of bacterial genera, which are known for the promotion of growth performance, immune modulation, and improvement of overall health in growing pigs, and improved the production of several microbial metabolites, such as *Lactobacillus*. PB1 and PB2 were recognized as safe in this study, as they did not promote any pathogenic microbiota, although they also did not seem to inhibit opportunistic pathogens as was observed with individual isolates. The blends did not negatively affect microbial metabolite production, although the effects of their individual supplementation were less prominent compared to their combination with casein. PB2 in combination with casein seemed to reduce putrefactive protein fermentation, as evidenced by the lower cumulative production of iso-butyrate and iso-valerate. We conclude that the combination of casein with phytogenic blends, in particular PB1, had the most beneficial effects among the studied supplements in vitro, although this data should be proven in further in vivo studies.

## Figures and Tables

**Table 1:** Products and their concentrations used for the supplementation of SLIM units.

Run	Product	Concentration
1	SIEM	Standard [14]
2	SIEM + casein	12 g casein *
3	SIEM + PB1	500 μL PB1 *
4	SIEM + PB1 + casein	12 g casein + 500 μL PB1 *
5	SIEM + PB2	500 μL PB2 *
6	SIEM + PB2 + casein	12 g casein + 500 μL PB2 *

* per day added to SIEM.

## Data Availability

All raw sequence data and metadata are available from the corresponding author upon reasonable request.

## Supplementary Materials

The following supporting information can be downloaded at: https://www.mdpi.com/article/10.3390/vetsci10120677/s1, Figure S1. Composition in the relative abundance of identified taxa at the phylum (a), class (b), order (c), and family (d) levels in samples from the swine large intestinal in vitro model after in-terventions. SIEM = standard ileal efflux medium, phytobiotic blends 1 and 2 = PB1 and PB2; Figure S2: Composition in the relative abundance of identified taxa at the phylum (a), class (b), order (c), family (d), and genus (e) levels in samples from the Swine Large Intestinal in vitro Model before interventions (at time-point 0 h); Figure S3: Results of differential abundance analysis at the phylum (a), class (b), order (c), and family (d) levels using MaAsLin2; Figure S4: Filtered relative abundance of genera that differ significantly after supplementation with casein, phytogenic blends 1 and 2 (PB1 and PB2) with or without casein, compared to standard ileal efflux medium (SIEM); Figure S5: Filtered relative abundance of families, that differ significantly after supplementation with casein, PB1, and PB2 with or without casein compared to SIEM; Figure S6: Filtered relative abundance of orders, that differ significantly after supplementation with casein, PB1, and PB2 with or without casein compared to SIEM; Figure S7: Filtered relative abundance of classes, that differ significantly after supplementation with casein, PB1, and PB2 with or without casein compared to SIEM; Figure S8: Filtered relative abundance of phyla, that differ significantly after supplementation with casein, PB1, and PB2 with or without casein compared to SIEM; Figure S9: Matrix with beta diversity Bray–Curtis distances between samples.
