# Supplementary material for: Modulation of Swine Gut Microbiota by Phytogenic Blends and High Concentrations of Casein in a Validated Swine Large Intestinal In Vitro Model
Source: Vet Sci. 2023 Nov 27;10(12):677. doi: 10.3390/vetsci10120677 (PMC10748322; doi:10.3390/vetsci10120677)
Supplement: Supplementary file 1 [file vetsci-10-00677-s001.zip › vetsci-2679575-supplementary.pdf]

veterinary sciences

MDPI

Supplementary figures

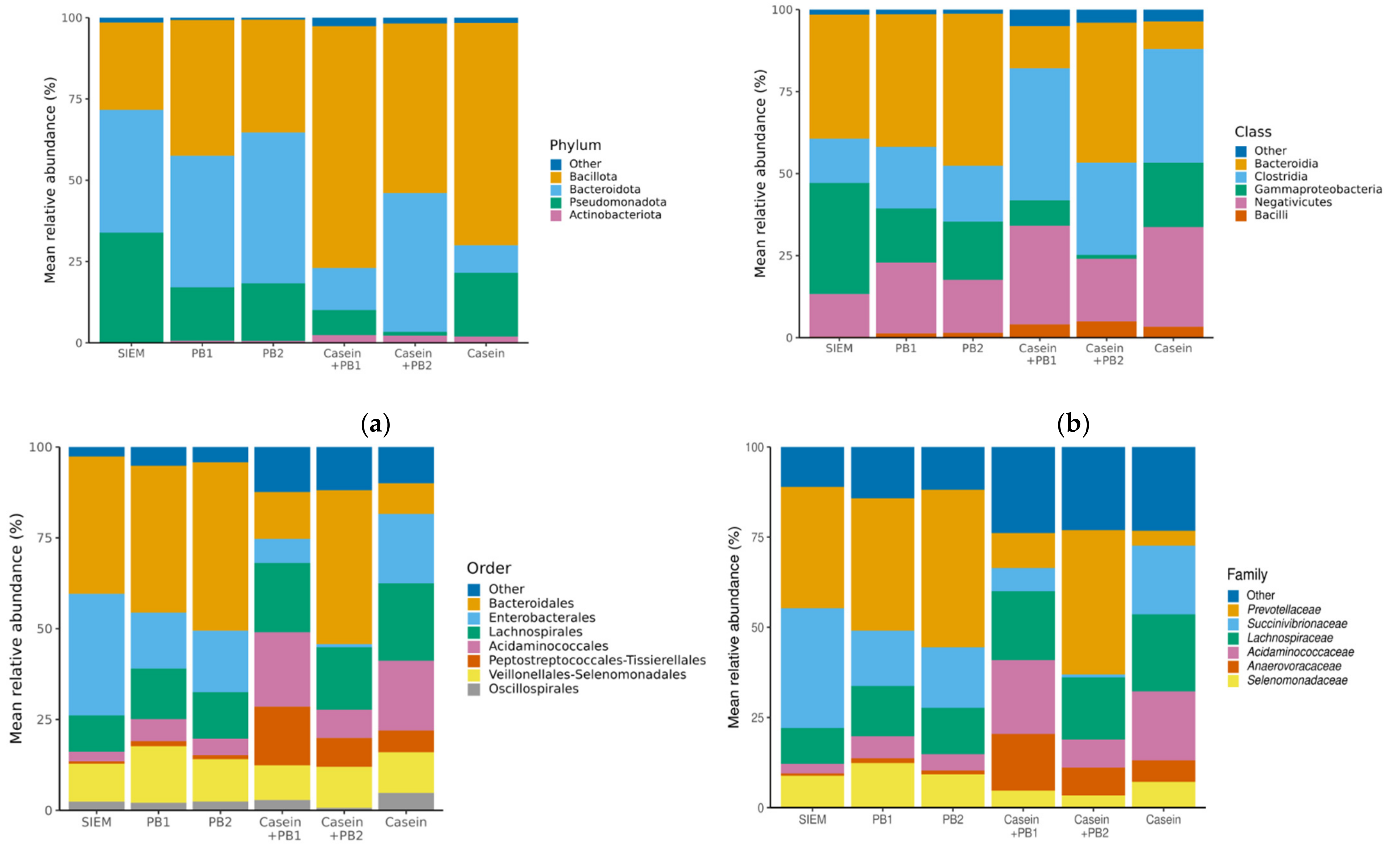

(a) (b)

(c) (d)

Figure S1. Composition in the relative abundance of identified taxa at the phylum (**a**), class (**b**), order (**c**), and family (**d**) levels in samples from the swine large intestinal *in vitro* model after interventions. SIEM = standard ileal efflux medium, phytobiotic blends 1 and 2 = PB1 and PB2.

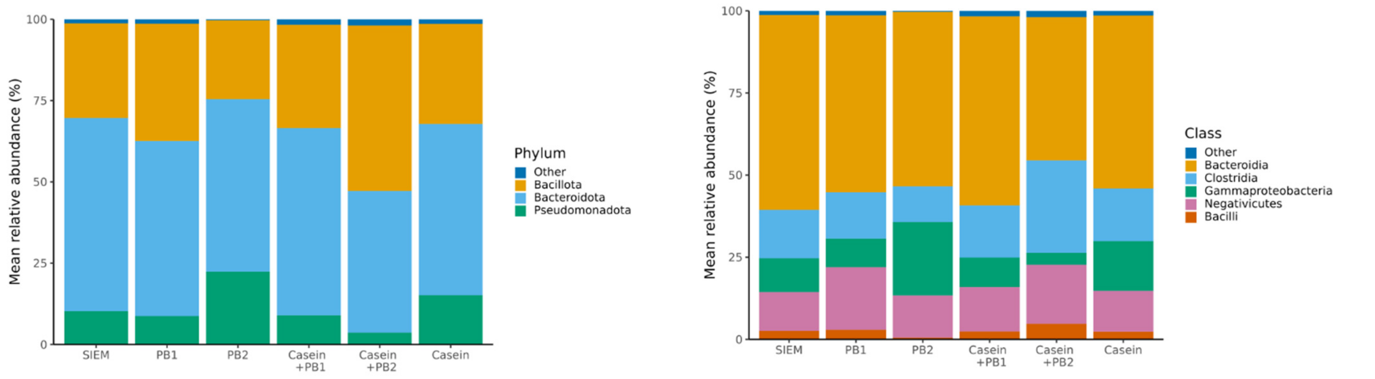

(a) (b)

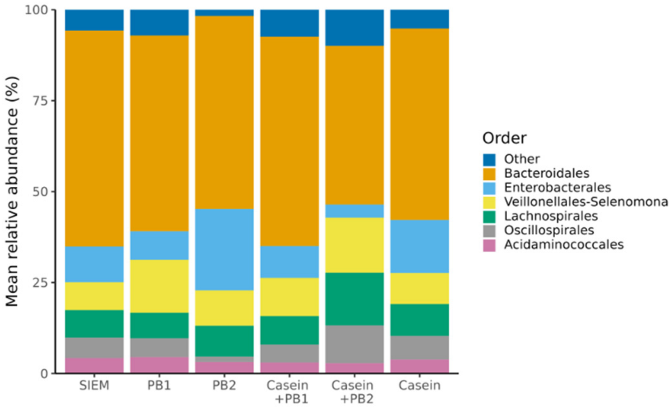

(**c**)

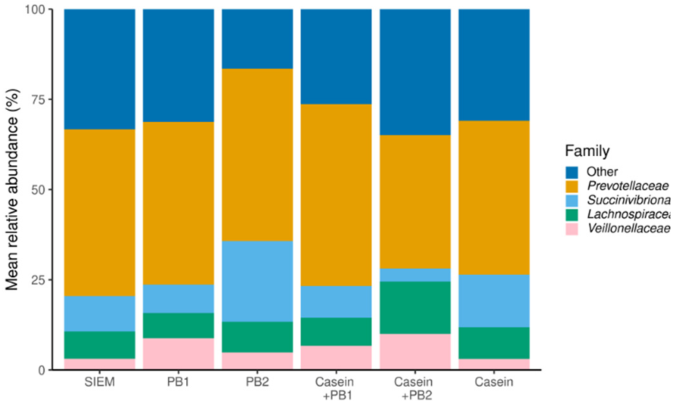

(**d**)

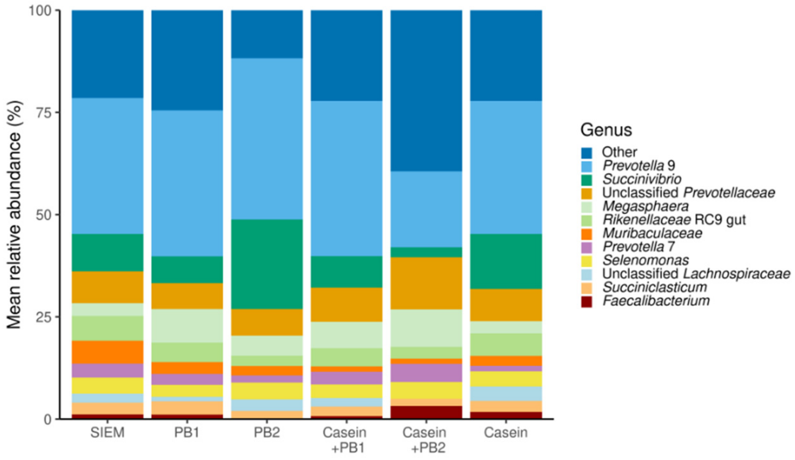

(**e**)

Figure S2. Composition in the relative abundance of identified taxa at the phylum (**a**), class (**b**), order (**c**), family (**d**), and genus (**e**) levels in samples from the swine large intestinal *in vitro* model before interventions (at time-point 0 h). SIEM = standard ileal efflux medium, phytobiotic blends 1 and 2 = PB1 and PB2.

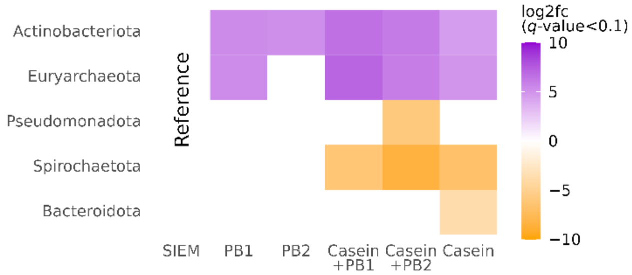

(**a**)

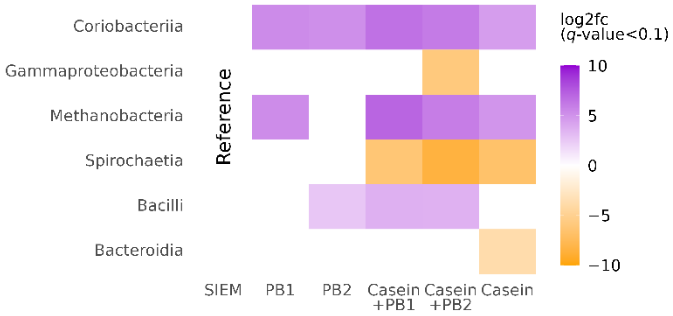

(**b**)

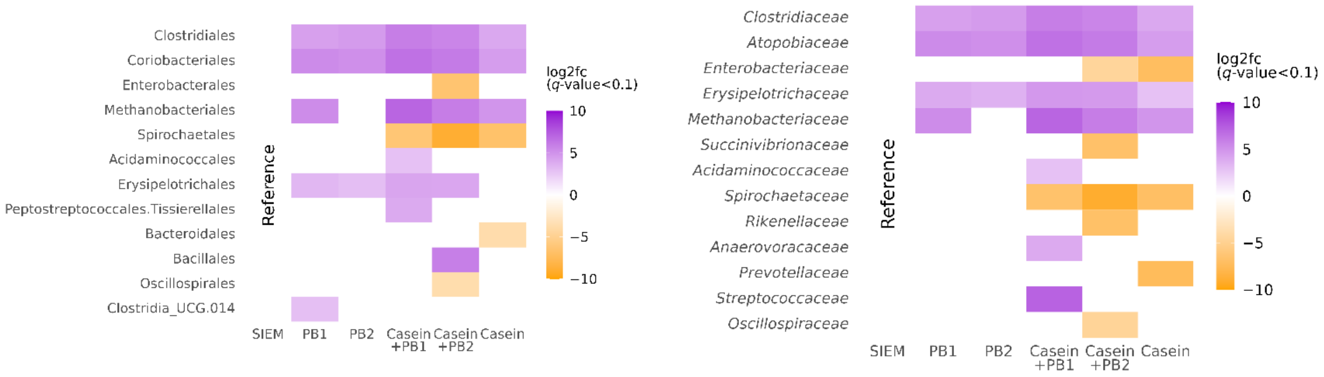

(c) (d)

Figure S3. Results of differential abundance analysis at the phylum (a), class (b), order (c), and family (d) levels using MaAsLin2. The heatmap represents a statistically significant log2-fold change (log2fc) of taxa in SLIM units supplemented with phytogenic blends 1 and 2 (PB1 and PB2), casein, and their combinations compared to control – standard ileal efflux medium (SIEM).

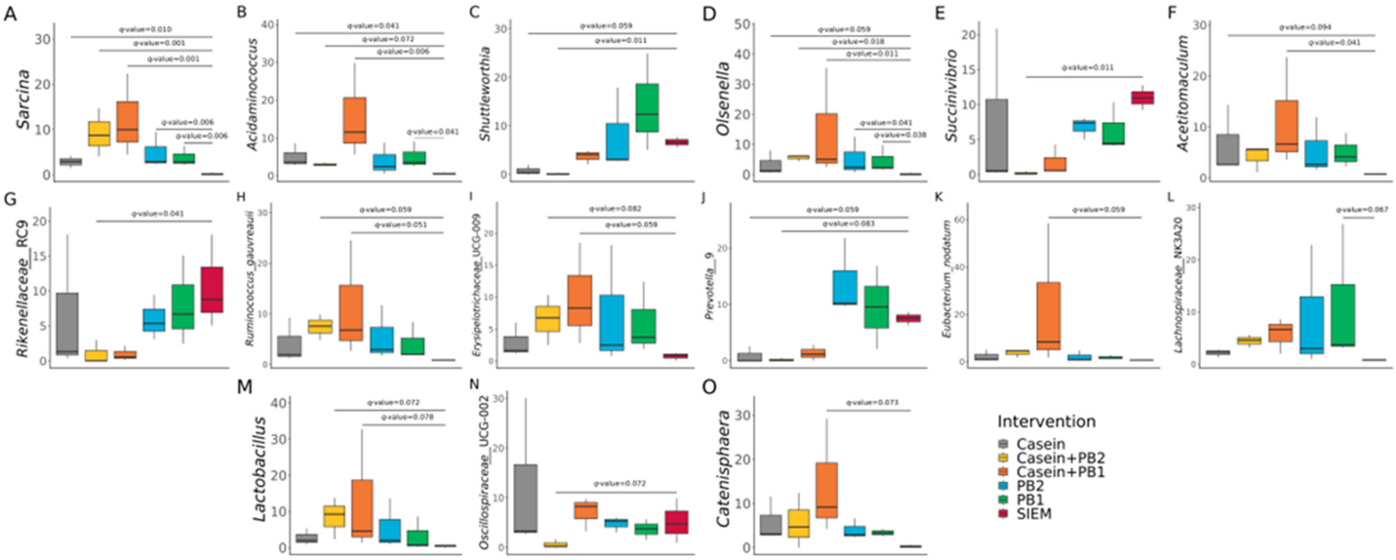

Figure S4. Filtered relative abundance of genera, that differ significantly after supplementation with casein, phytogenic blends 1 and 2 (PB1 and PB2) with or without casein compared to standard ileal efflux medium (SIEM): (A) *Sarcina*, (B) *Acidaminococcus*, (C) *Shuttleworthia*, (D) *Olsenella*, (E) *Succinivibrio*, (F) *Acetitomaculum*, (G) *Rikenellaceae_RC9*, (H) *Ruminococcus._gauvreauii*, (I) *Erysipelotrichaceae_UCG.009*, (J) *Prevotella_9*, (K) *Eubacterium._nodatum*, (L) *Lachnospiraceae_NK3A20*, (M) *Lactobacillus*, (N) *UCG.002*, (O) *Catenisphaera*. Q-values were calculated with MaAsLin2 following Benjamini-Hochberg false discovery rate correction.

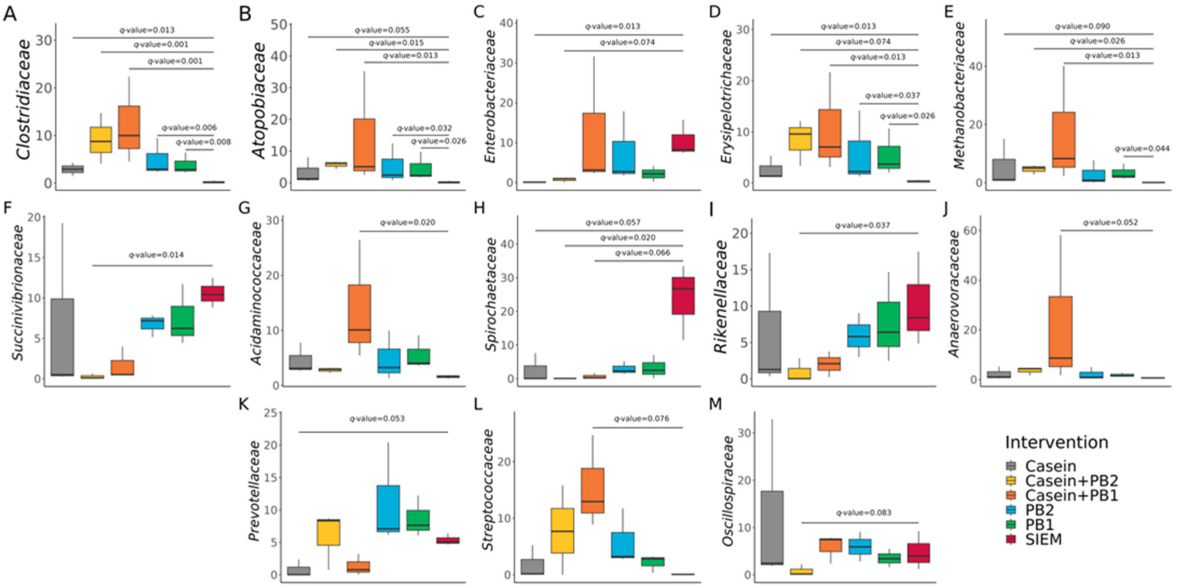

Figure S5. Filtered relative abundance of families, that differ significantly after supplementation with casein, phytogenic blends 1 and 2 (PB1 and PB2) with or without casein compared to standard ileal efflux medium (SIEM): (A) *Clostridiaceae*, (B) *Atopobiaceae*, (C) *Enterobacteriaceae*, (D) *Erysipelotrichaceae*, (E) *Methanobacteriaceae*, (F) *Succinivibrionaceae*, (G) *Acidaminococcaceae*, (H) *Spirochaetaceae*, (I) *Rikenellaceae*, (J) *Anaerovoracaceae*, (K) *Prevotellaceae*, (L) *Streptococcaceae*, (M) *Oscillospiraceae*. Q-values were calculated with MaAsLin2 following Benjamini-Hochberg false discovery rate correction.

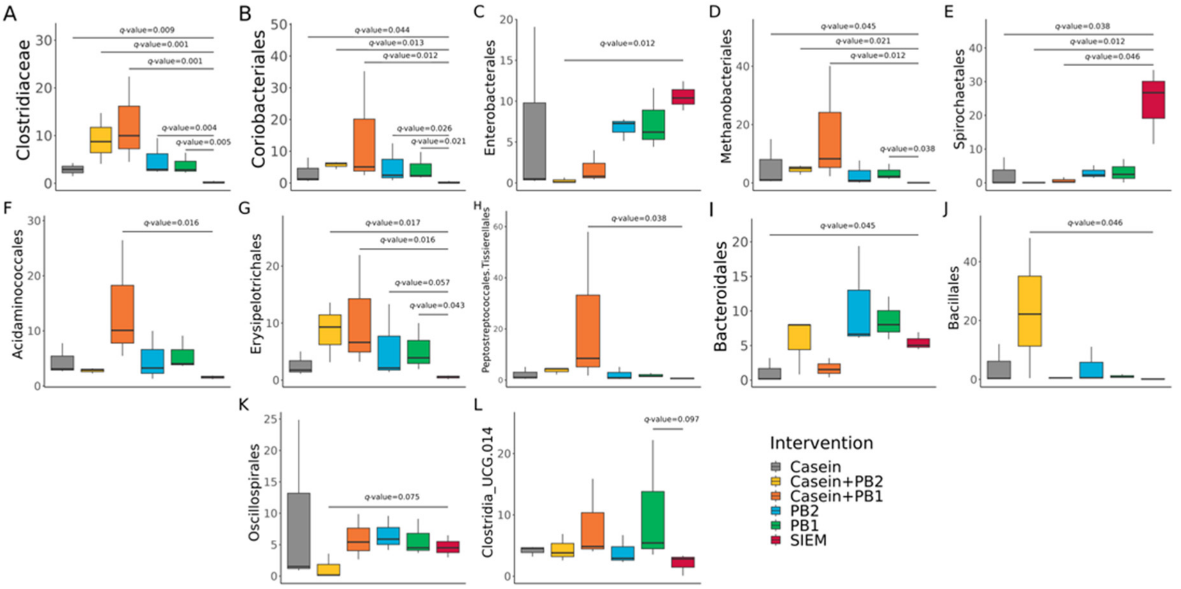

Figure S6. Filtered relative abundance of orders, that differ significantly after supplementation with casein, phytogenic blends 1 and 2 (PB1 and PB2) with or without casein compared to standard ileal efflux medium (SIEM): (A) Clostridiales, (B) Coriobacteriales, (C) Enterobacterales, (D) Methanobacteriales, (E) Spirochaetales, (F) Acidaminococcales, (G) Erysipelotrichales, (H) Peptostreptococcales.Tissierellales, (I) Bacteroidales, (J) Bacillales, (K) Oscillospirales, (L) Clostridia_UCG.014. Q-values were calculated with MaAsLin2 following Benjamini-Hochberg false discovery rate correction.

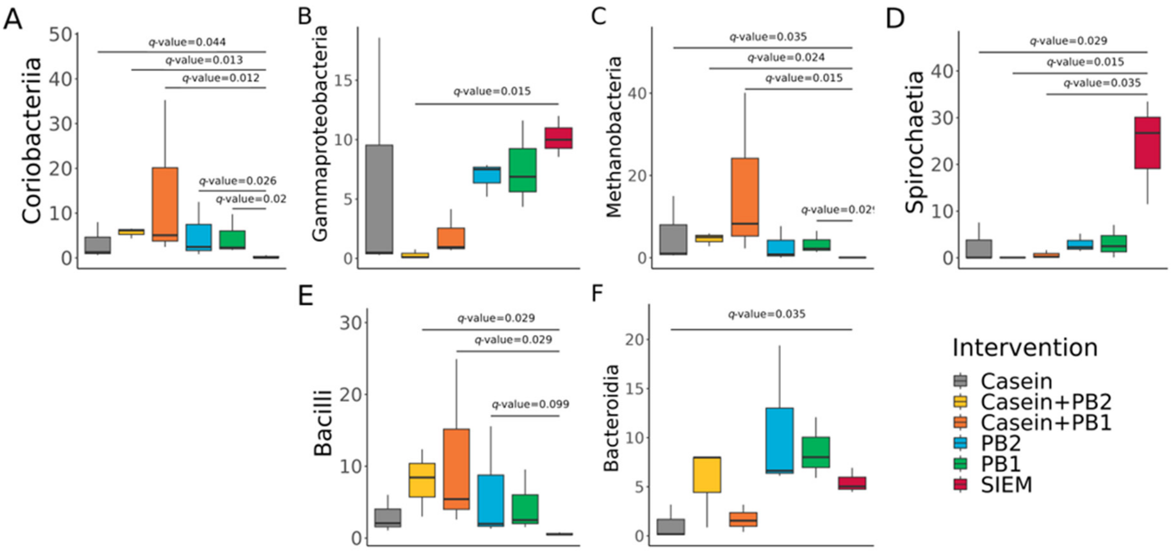

Figure S7. Filtered relative abundance of classes, that differ significantly after supplementation with casein, phytogenic blends 1 and 2 (PB1 and PB2) with or without casein compared to standard ileal efflux medium (SIEM): (A) Coriobacteriia, (B) Gammaproteobacteria, (C) Methanobacteria, (D) Spirochaetia, (E) Bacilli, (F) Bacteroidia. Q-values were calculated with MaAsLin2 following Benjamini-Hochberg false discovery rate correction.

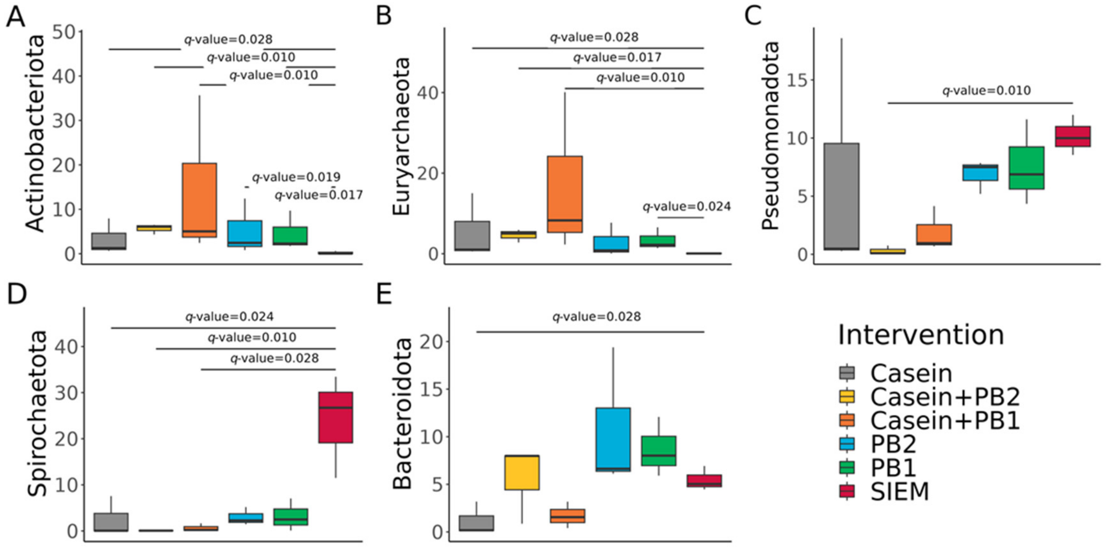

Figure S8. Filtered relative abundance of phyla, that differ significantly after supplementation with casein, phytogenic blends 1 and 2 (PB1 and PB2) with or without casein compared to standard ileal efflux medium (SIEM): (A) Actinobacteriota, (B) Euryarchaeota, (C) Proteobacteria, (D) Spirochaetota, (E) Bacteroidota. Q-values were calculated with MaAsLin2 following Benjamini-Hochberg false discovery rate correction.

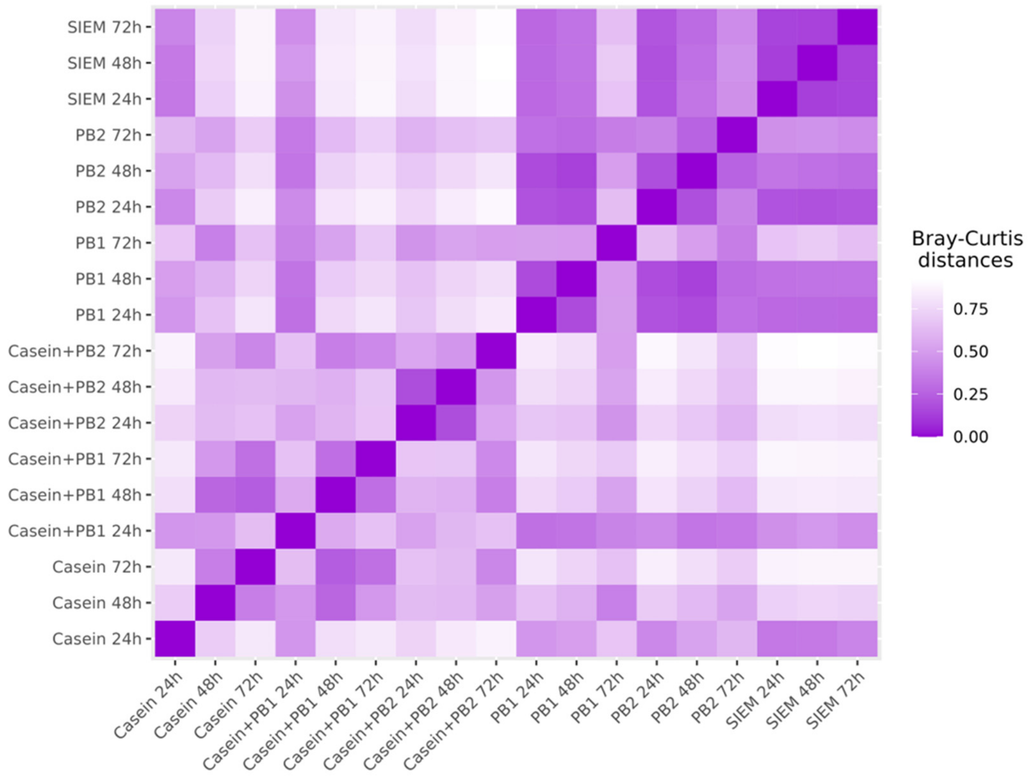

Figure S9. Matrix with beta diversity Bray-Curtis distances between samples. The dark purple color represents a short distance between samples (indicating similar microbiota composition), light purple color represents a long distance between samples (indicating larger differences in microbiota composition).
